# Draft Genome Sequence of the Prazosin-Degrading *Bacillus* sp. Strain PR5, Isolated from a River Receiving Hospital and Urban Wastewater in Malaysia

**DOI:** 10.1128/MRA.00025-21

**Published:** 2021-03-18

**Authors:** Nurul Syazwani Ahmad Sabri, Nur’Aqilah Farhanah Mohd Mohsi, Atiqqoh Apandi, Nurtasbiyah Yusof, Megat Johari Megat Mohd Noor, Fazrena Nadia Md Akhir, Norio Sugiura, Motoo Utsumi, Nor’azizi Othman, Zuriati Zakaria, Hirofumi Hara

**Affiliations:** a Department of Chemical and Environmental Engineering, Malaysia-Japan International Institute of Technology, Universiti Teknologi Malaysia, Kuala Lumpur, Malaysia; b Graduate School of Life and Environmental Science, University of Tsukuba, Tsukuba, Ibaraki, Japan; c Department of Mechanical Precision Engineering, Malaysia-Japan International Institute of Technology, Universiti Teknologi Malaysia, Kuala Lumpur, Malaysia; University of Delaware

## Abstract

We report the complete genome sequence of *Bacillus* sp. strain PR5, isolated from a river receiving hospital and urban wastewater in Malaysia, which demonstrated a high capability for degrading prazosin. This genome sequence of 4,525,264 bp exhibited 41.5% GC content, 4,402 coding sequences, and 32 RNAs.

## ANNOUNCEMENT

Prazosin is a drug that is mainly prescribed to treat hypertension and posttraumatic stress disorder (PTSD) (1–4). Traces of these pharmaceutical compounds have been detected in drinking water and sewage treatment plants at 5 ng/L and 117 ng/L, respectively (5, 6). Accumulation of prazosin in the environment is detrimental to aquatic ecosystems and humans (7, 8). Beneficial microbes serve as a potential means of removing prazosin contaminants from the environment. In this study, we isolated *Bacillus* sp. strain PR5 from a river receiving hospital and urban wastewater that showed prazosin-degrading activity. Here, we report the draft genome sequence of this strain.

A water sample was collected from the Pantai River, located near Pantai Hospital in Kuala Lumpur, Malaysia (3°07′01.5″N, 101°39′51.4″E). Upon thorough filtration and dilution, a heterogeneous solution was cultured on solidified minimal salt medium (MSM) with the addition of 0.1 M prazosin (9, 10) as the sole nitrogen source until a pure culture was obtained (7). The pure culture was then grown in Luria-Bertani (LB) broth at 30°C with constant agitation at 160 rpm until the exponential phase was reached. Genomic DNA was extracted using the HiYield genomic DNA minikit (RBC BioScience, Taiwan) prior to sequencing using the Ion S5XL system (Thermo Fisher Scientific). A 400-bp library was constructed using the Ion Xpress Plus fragment library kit (Thermo Fisher Scientific) according to the manufacturer’s protocol and quantified using an Agilent 2100 bioanalyzer. The library was diluted prior to template preparation using the Ion Chef system (Thermo Fisher Scientific) loaded with an Ion 530 chip, followed by sequencing for 4 h. Torrent Suite software (Thermo Fisher Scientific) was used for raw data analysis, alignment, and variant calling. The total number of sequencing reads obtained was 3,136,917 reads with an average read length of 361 bp. Short reads from the Ion S5XL system were quality trimmed and assembled using CLC Genomics Workbench software version 11.0.1 (CLC bio, Aarhus, Denmark). The reads were trimmed with the following parameters: quality score limit, 0.05; discarded reads, <400 nucleotides; and maximum number of ambiguous nucleotides, 2. Default parameters were used for the assembly.

The assembly yielded 252 contigs for a total genome size of 4,525,264 bp and an *N*_50_ value of 39,349 bp. The GC content of the genome is 41.5%. Annotation using the NCBI Prokaryotic Genome Annotation Pipeline (PGAP) predicted 4,402 coding sequences, 10 rRNA genes, and 18 tRNAs (11). Using the Rapid Annotations using Subsystems Technology (RAST) server, 24 enzymes, including cytochrome P450 (cyp450), which belongs to the nitrogen metabolism category, were predicted to be involved in metabolizing prazosin compounds (12–14). In addition, 21 enzymes were annotated as responsible for the conversion of aromatic compounds, including decarboxylases and hydroxylases. The genome information of *Bacillus* sp. strain PR5 will provide insight into the biodegradation of prazosin, including the structure of quinazoline, which will play a significant role in the bioremediation of xenobiotic compounds from the environment.

### Data availability.

The complete genome sequence of *Bacillus* sp. strain PR5 was deposited at GenBank under BioProject number PRJNA489759 and BioSample number SAMN16830461 with SRA accession number SRS7801199 and assembly number GCA_016458605.1.
